# Antibacterial Activity of Medicinal Plants Against Pathogens causing Complicated Urinary Tract Infections

**DOI:** 10.4103/0250-474X.54279

**Published:** 2009

**Authors:** Anjana Sharma, S. Chandraker, V. K. Patel, Padmini Ramteke

**Affiliations:** Bacteriology Laboratory, Department of Post Graduate Studies and Research in Biological Sciences, R. D. University, Jabalpur-482 001, India

**Keywords:** Urinary tract infection (UTI), multidrug resistant, antibacterial activity, Indian medicinal plants

## Abstract

Seventeen Indian folklore medicinal plants were investigated to evaluate antibacterial activity of aqueous, ethanol and acetone extracts against 66 multidrug resistant isolates of major urinary tract pathogens (*Escherichia coli, Klebsiella pneumoniae, Pseudomonas aeruginosa* and *Enterococcus faecalis*) by disc diffusion method. Ethanol extract of *Zingiber officinale* and *Punica granatum* showed strong antibacterial activity against *Escherichia coli.* Ethanol extracts of *Terminalia chebula* and *Ocimum sanctum* exhibited antibacterial activity against *Klebsiella pneumoniae*. Ethanol extract of *Cinnamomum cassia* showed maximum antibacterial activity against *Pseudomonas aeruginosa* while ethanol extract of *Azadirachta indica* and *Ocimum sanctum* exhibited antibacterial activity against *Enterococcus faecalis*. The results support the folkloric use of these plants in the treatment of urinary tract infections by the tribals of Mahakoshal region of central India.

Urinary tract infections (UTI) are most common form of bacterial infections, affecting people throughout their lifespan[1,2]. The pathogenesis of complicated and uncomplicated UTI is complex and influenced by many host biological behavioral factors and properties of the infecting uropathogens. Leading etiological agents of UTI's include *Escherichia coli*, *Candida albicans*, *Enterococcus faecalis*, *Pseudomonas aeruginosa*, *Klebsiella pneumoniae* and *Proteus mirabilis*[3]. The incidence of acute uncomplicated UTI is estimated to exceed 0.5 episodes per annum among women between 18-30 y[4]. Despite the existence of potent antibiotics, resistant or multiresistant strains are continuously appearing, imposing the need for a permanent search and development of new drugs. For Centuries plants have been used throughout the world as drugs and remedies for various diseases[5]. These drugs serve as prototype to develop more effective and less toxic medicines. Hence, an attempt has been made to evaluate antibacterial activity of seventeen folklore medicinal plants used by tribals in Mahakoshal region of central India against urinary tract pathogens.

Plant parts were collected on the basis of the information provided in the ethanobotanical survey of India and local medicine men of Mahakoshal region (MP), India. Each specimen/plant material was labeled, numbered, annoted with the date of collection, locality and their medicinal uses were recorded (Table 1). Fresh plant material was washed thoroughly, air dried and then homogenized to fine powder and stored in airtight containers[6].

**TABLE 1:** SELECTED INDIAN MEDICINAL PLANTS FOR ANTIBACTERIAL ACTIVITY

Plant Species and common name	Family	Part used	Medicinal uses
*Z. officinale* (*Adrak*)	Zingiberaceae	Rhizome	Analgesic, sedative, antipyretic and antibacterial
*C. cassia* (*Dalchini*)	Lauraceae	Bark	Antibacterial, circulatory, respiratory, uterotonic and stomachic
*T. chebula* (*Harrah*)	Combretaceae	Fruit	Laxative, stomachic, tonic and alternative
*P. ovata* (*Isabgol*)	Plataginaceae	Husk	Constipation, colitis, irritable bowel, cystitis
*A. nilotica* (*Babool*)	Fabaceae	Leaf	Treating premature ejaculation
*P. anisum* (*Saunph*)	Apiaceae	Seed	Antiseptic, antispasmodic, aromatic, carminative, digestive, galactogogue, pectoral, stimulant
*C. asiatica* (*Brahmi*)	Apiaceae	Stem	Tonic, sedative, alterative
*O. sanctum* (*Tulsi*)	Laminaceae	Leaf	Cures cough, cold, chronic, catarrh, nausea, respiratory problem, skin diseases, antibacterial
*A. indica* (*Neem*)	Meliaceae	Fruit	Skin disease, blood disorder, antibacterial
*P. fraternus* (*Bhumiamla*)	Euphorbiaceae	Leaf	Jaundice, liver disease, fever, genitourinary disease, edema
*C. sativum* (*Dhana*)	Apiaceae	Seed	Flatulence, colic, joint pain, antiseptic
*A. indicum* (*Atibala*)	Malvaceae	Stem	Demulcent, aphrodisiac, laxative, astringent and diuretic, expectorant, antiinflammatory and analgesic
*P. granatum* (*Anar*)	Lythraceae	Seed	Anthelminthic (esp. tapeworm), diarrhea, dyspepsia
*S. cumini* (*Jamun*)	Myrtaceae	Bark	Astringent, stomachic, carminative, antiscorbutic, diuretic
*C. scariosus* (*Nagarmotha*)	Cyperaceae	Root	Astringent, diaphoretic, desiccant, cordial and stomachic
*A. paniculata* (*Kalmegh*)	Acanthaceae	Bark	Laxative, vulnerary, antipyretic, antiperiodic, antiinflammatory, expectorant, depurative, antibacterial
*M. indica* (*Aam*)	Anacardiaceae	Leaf	Supplement of sexual potency, antiallergic, hypoglycaemic and antidiabetic

Plant material (10 g) was extracted with 100 ml distilled water by subjecting it slow heat for 6 h; filtered through muslin cloth and centrifuged at 5000×g for 15 min. The supernatant was collected and concentrated to one-fourth of the total volume[7]. Organic solvent extraction of the plant materials were prepared according to the method described by Nair *et al.*[7] with some modifications. 10 g of plant material was crushed and blended into powder using an electric blender with each organic solvent i.e ethanol and acetone separately. The blended material was subjected to agitation on rotary shaker (190-220 rpm) for 30 min, filtered with muslin cloth; centrifuged (5000×g for 15 min), and concentrated to one-fourth of the total volume.

The test organisms included the gram-positive *E. faecalis* (7 isolates) and gram-negative *E. coli* (34 isolates), *K. pneumoniae* (15 isolates), and *P. aeruginosa* (10 isolates). All the 66 UTI isolates were obtained from the bacterial germplasm culture collection, Bacteriology laboratory, R. D. University, Jabalpur, India. The bacteria were grown in the nutrient broth at 37° and maintained on nutrient agar slants at 4°.

*In vitro* antibacterial activity was examined for aqueous, ethanol and acetone extracts from 17 different traditionally used medicinal plants. Antimicrobial activities of these extracts were evaluated by Agar disc diffusion method[8]. For all the bacterial strains, overnight cultures grown in broth were adjusted to an inoculum size of 10^6^ CFU/ml for inoculation of the agar plates[9]. A lawn of UTI bacterial culture[10] was made on Mueller Hinton agar plates and sterile filter paper discs soaked with 10 μl (concentration 50 mg/ml) each of aqueous, ethanol and acetone extract was placed on it. Disc soaked in distilled water, ethanol and acetone served as control. Following an incubation period of 24 h at 37°, antibacterial activity was evaluated by inhibition zones of bacterial growth. The results are represented as average zone of inhibition of all the isolates of individual species.

Fifty-one aqueous, ethanol and acetone extracts of the plant belonging to 17 families were tested against gram-positive and gram-negative urinary tract pathogenic bacteria. Ethanolic extract showed considerably more activity than the acetone and aqueous extract. Maximum antibacterial activity was shown by ethanol extract of *Z. officinale*, *P. granatum* and acetone extract of *T. chebula* against *E. coli* with the inhibition of 14 mm zone size (fig. 1). Similar results were observed by previous scientist against pathogenic bacteria[11–14]. Ethanol extracts of *T. chebula* and *O. sanctum* were found to be active with the inhibition of 16 mm against *K. pneumoniae* (fig. 2) while ethanol extract of *C. cassia* showed significant activity with 18 mm of clear inhibition zone against *P. aeruginosa* (fig. 3). Previous reports from china have revealed that various parts of *C. cassia* such as the fruit, oil, inner bark and leafy twigs possessed good antimicrobial activity. The ethanol extract of *C. asiatica* and *O. sanctum* show broad-spectrum activity against *E. faecalis* with the inhibition zone of 19 mm (fig. 4). Best antimicrobial activity was found in case of ethanol extract of *Z. officinale, P. granatum, T. chebula, O. sanctum, C. cassia*, *C. asiatica* and acetone extract of *T. chebula* against multidrug resistant UTI pathogens. This probably explains the use of these plants by indigenous people against a number of infections since generations. The plants studied here had shown that they are potentially rich in antimicrobial compounds and have also been extensively used by the tribals. The millenarian use of these plants in folk medicine suggests that they represent an economic and safe alternative for treatment of Urinary Tract Infections.

**Fig. 1:**
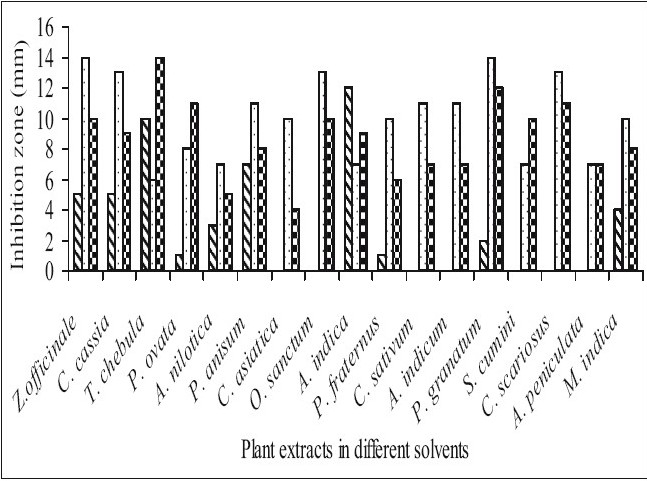
Antibacterial activity of plant extracts against *Escherichia coli*. Antibacterial activity of aqueous (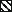), ethanol (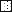) and acetone (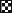) extracts of plants against *Escherichia coli*

**Fig. 2:**
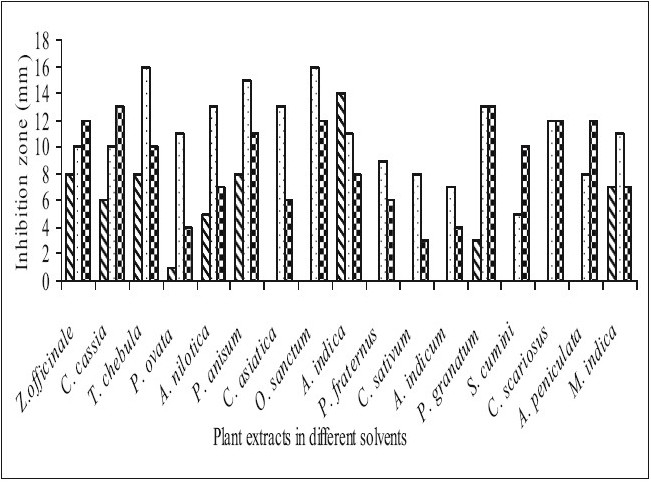
Antibacterial activity of plant extracts against *Klebsiella pneumoniae*. Antibacterial activity of aqueous (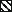), ethanol (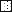) and acetone (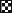) extracts of plants against *Klebsiella pneumoniae*

**Fig. 3:**
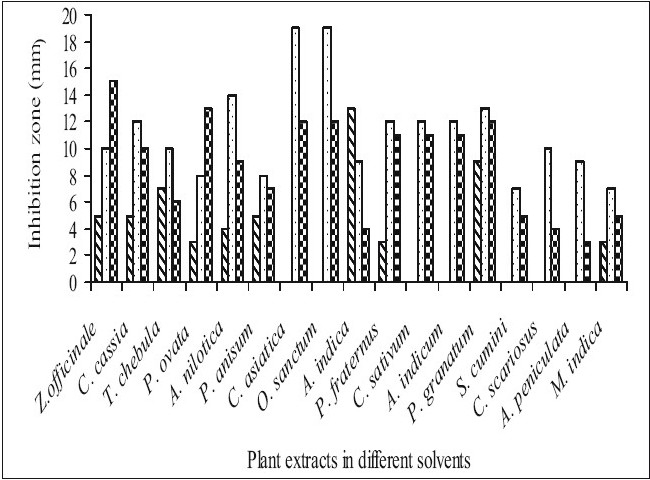
Antibacterial activity of plant extracts against *Pseudomonas aeruginosa*. Antibacterial activity of aqueous (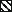), ethanol (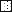) and acetone (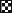) extracts of plants against *Pseudomonas aeruginosa*

**Fig. 4:**
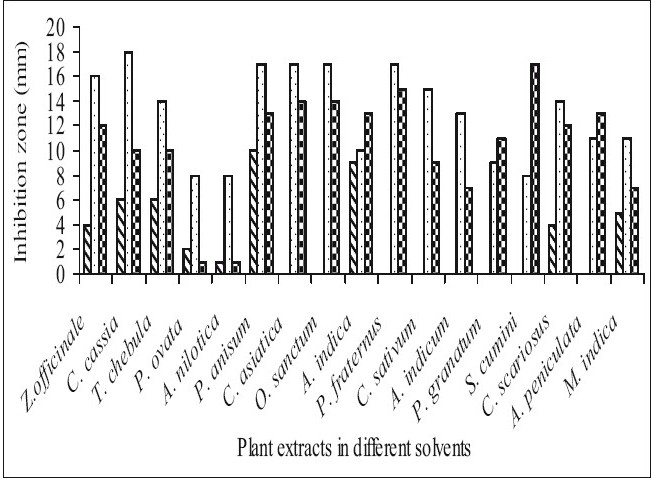
Antibacterial activity of plant extracts against *Enterococcus faecalis*. Antibacterial activity of aqueous (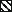), ethanol (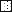) and acetone (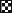) extracts of plants against Enterococcus faecalis
